# Non-Oxidative Propane Dehydrogenation on CrO_x_-ZrO_2_-SiO_2_ Catalyst Prepared by One-Pot Template-Assisted Method

**DOI:** 10.3390/molecules27186095

**Published:** 2022-09-18

**Authors:** Elena V. Golubina, Igor Yu. Kaplin, Anastasia V. Gorodnova, Ekaterina S. Lokteva, Oksana Ya. Isaikina, Konstantin I. Maslakov

**Affiliations:** Department of Chemistry, Lomonosov Moscow State University, Leninskie Gory, 1, Bld. 3, 119991 Moscow, Russia

**Keywords:** propane dehydrogenation, propylene, mixed oxide catalysts, chromia, zirconia, one-pot synthesis

## Abstract

A series of CrO_x_-ZrO_2_-SiO_2_ (CrZrSi) catalysts was prepared by a “one-pot” template-assisted evaporation-induced self-assembly process. The chromium content varied from 4 to 9 wt.% assuming Cr_2_O_3_ stoichiometry. The catalysts were characterized by XRD, SEM-EDX, temperature-programmed reduction (TPR-H_2_), Raman spectroscopy, and X-ray photoelectron spectroscopy. The catalysts were tested in non-oxidative propane dehydrogenation at 500–600 °C. The evolution of active sites under the reaction conditions was investigated by reductive treatment of the catalysts with H_2_. The catalyst with the lowest Cr loading initially contained amorphous Cr^3+^ and dispersed Cr^6+^ species. The latter reduced under reaction conditions forming Cr^3+^ oxide species with low activity in propane dehydrogenation. The catalysts with higher Cr loadings initially contained highly dispersed Cr^3+^ species stable under the reaction conditions and responsible for high catalyst activity. Silica acted both as a textural promoter that increased the specific surface area of the catalysts and as a stabilizer that inhibited crystallization of Cr_2_O_3_ and ZrO_2_ and provided the formation of coordinatively unsaturated Zr^4+^ centers. The optimal combination of Cr^3+^ species and coordinatively unsaturated Zr^4+^ centers was achieved in the catalyst with the highest Cr loading. This catalyst showed the highest efficiency.

## 1. Introduction

Olefins are important chemical intermediates used in the large-scale production of polymers, rubber, and higher alcohols, and in the smaller scale production of other chemicals. Propylene is mainly consumed in the production of polypropylene that is a raw material for a wide range of products—plastics, propylene oxide, isopropyl alcohol, acrylonitrile, cumene, acrylic acid, etc. [1]. Refinery streams contain recoverable fractions of propylene and can serve as its source [2]. However, usually, propylene is produced by steam cracking of propane or higher hydrocarbons, or as a by-product of fluid catalytic cracking (FCC), but with a low yield. Therefore, new sources of propylene are required. Significant amounts of propane are found in natural and shale gases [3]. That is why the development of the catalysts for the direct dehydrogenation of propane to propylene is an important and urgent task.

There are several routes of propane dehydrogenation that can be divided into non-oxidative and oxidative ones. Oxidative dehydrogenation has an advantage of being exothermic and irreversible [4,5]. This reaction is thermodynamically favorable in any alkane conversion and alkene yield. At the same time, a major drawback of oxidative dehydrogenation is the inevitable oxidation of propane and propylene to oxygen-containing organic molecules and CO_x_, which leads to both the loss of hydrocarbon feedstock and CO_2_ emission that contributes to significant global climate change [6]. More selective VO_x_/SBA-15 catalysts [7] and mild oxidizing agents, for example, CO_2_, [6] and N_2_O [8] were proposed to improve propene selectivity.

An alternative approach for propane conversion is non-oxidative propane dehydrogenation (PDH). In contrast to oxidative dehydrogenation, the efficiency of PDH is hampered by the thermodynamics of this process. The equilibrium conversion decreases when increasing the partial pressure of propane [4]. Propane dehydrogenation is a highly endothermic process. The enthalpy of the C_3_H_8_ → C_3_H_6_ + H_2_ reaction is 124 kJ mol^−1^ [9]. High temperatures are required to achieve the high propylene yield, but they also favor thermal cracking reaction that leads to coke formation and deactivation of the catalyst, making necessary its regeneration. Sometimes co-feeding of H_2_ is used to suppress coke formation, but this has a negative effect on the conversion [4]. All these facts make it necessary to develop highly efficient coke-resistant catalysts of propane dehydrogenation with high propane conversion and propylene selectivity.

The nature and structure of the active sites in alkane dehydrogenation catalysts have been widely studied. Alumina-supported chromium oxide (CATOFIN^®^ from Lummus Technology Inc., Houston, TX, USA) and Pt-Sn/Al_2_O_3_ (Oleflex from UOP, Des Plaines, IL, USA) are modern industrial catalysts of non-oxidative PDH. They demonstrate reasonable propane to propylene conversions [4,9]. In both cases, coking is an undesirable side process. An additional disadvantage of chromium-containing industrial catalysts is a high chromium content, which can pose a threat to human health due to the presence of toxic Cr^6+^ compounds. V [10,11], Co [12,13], MoO_x_/Al_2_O_3_ [14], Rh, Rh, Ru, Pt or Ir [15], Rh-Sn [16], PtGa/Al_2_O_3_ [17], and Co-V oxide [18] catalysts are also active in PDH.

The high activity of amorphous Cr_2_O_3_ particles in chromium-containing catalysts was demonstrated [19,20]. However, at high reaction temperatures these particles can crystallize to form α-Cr_2_O_3_ that has a lower activity in PDH [19]. Therefore, stabilization of amorphous Cr_2_O_3_ phase is very important. Surface Cr^3+^ and coordinatively unsaturated Cr^2+^ species were proposed to be the active sites of the dehydrogenation reaction, while Cr^6+^ or Cr^5+^ species are usually considered as inactive in this process [1]. In general, Cr^6+^, Cr^3+^, and Cr^2+^ species can exist in different ratios on the catalyst surface after various treatments during catalyst synthesis. Earlier, a series of CrO_x_/SBA-1 catalysts containing chromium in different oxidation states (Cr^6+^, Cr^5+^, and Cr^3+^ in crystalline Cr_2_O_3_) was investigated in non-oxidative PDH [19]. It was shown that under the reaction conditions, Cr^6+^ species were rapidly (within the first 10 min of the test) reduced to Cr^3+^/Cr^2+^ ones. Thus, controlling the oxidation state of chromium is important for the catalyst efficiency.

Increasing the activity of PDH catalysts by introducing modifiers and tuning metal–support interaction has been proposed in recent years. Zirconia-based catalysts are known for their high performance in alkane dehydrogenation. A promising way to increase the activity of chromium catalysts in PDH is the co-existence of CrO_x_ and coordinatively unsaturated Zr^4+^ (Zr_cus_^4+^) sites as was demonstrated for CrZrO_x_, CrO_x_/LaZrO_x_, and CrO_x_/Al_2_O_3_ catalysts [20]. Zr_cus_^4+^ ions can be generated by doping of zirconia with non-noble elements [11,21]. The formation of well-dispersed Zr_cus_^4+^ ions is vital for high catalyst activity [22]. The important role of Zr_cus_^4+^ sites was shown for LaZrO_x_, Ru/LaZrO_x_, and Rh/LaZrO_x_ systems [21]: the catalyst efficiency was more dependent on the presence of Zr_cus_^4+^ sites than on Ru or Rh ones. It was demonstrated that the ratio between the numbers of chromium-containing active sites and coordinatively unsaturated zirconium sites is very important for ensuring high catalyst efficiency [23]. Crystallinity of zirconia is another important factor affecting the catalyst efficiency. As the formation of Zr_cus_^4+^ sites depends on the zirconia crystallinity, it also influences the catalyst efficiency.

The low specific surface area of zirconium oxide can be addressed by its modification. For example, the specific surface area of LaZrO_x_ PDH catalysts reached about 100 m^2^/g in contrast to 38 m^2^/g of pure ZrO_2_ [20]. In the same way, the modification of ZrO_2_ with chromium increased its specific surface area from 24 m^2^/g up to 107 m^2^/g for Cr_10_Zr_90_O_x_, but a lower value of 77 m^2^/g was observed for a higher Cr content of 20 mol.% [23]. Supporting on silica can further increase the specific surface area of CrZrO_x_ oxide up to 220–240 m^2^/g [24].

This work focuses on the synthesis of CrO_x_-ZrO_2_-SiO_2_ catalysts with large specific surface area and different chromium loadings using the “one-pot” template-assisted evaporation-induced self-assembly process. In these catalysts, CrO_x_ is an active component, while ZrO_2_ serves as a source of coordinatively unsaturated Zr^4+^ sites, and SiO_2_ is a structuring agent that increases the specific surface area and provides high dispersion of zirconia. The catalysts are tested in non-oxidative propane dehydrogenation at 500–600 °C. The effect of chromium loading on the catalyst efficiency is analyzed.

## 2. Results

### 2.1. Catalyst Characterization

The physicochemical characteristics of the catalysts are presented in Table 1. The chromium content measured by AAS was close to the expected values.

All samples were mesoporous with similar average pore sizes (about 3.7 nm) calculated from the BJH pore size distributions. The specific surface areas (S_BET_) of all the catalysts are close and significantly larger than the typical values for pure ZrO_2_ [25]. The catalysts of the similar chemical composition prepared by the impregnation method showed smaller S_BET_ [20,24]. The increased S_BET_ values of the synthesized catalysts resulted from the modification of zirconia with SiO_2_ and the use of an organic CTAB template during synthesis. The addition of chromium oxide to the ZrSi catalyst during the “one-pot” synthesis did not significantly change its textural characteristics.

**Figure 1:**
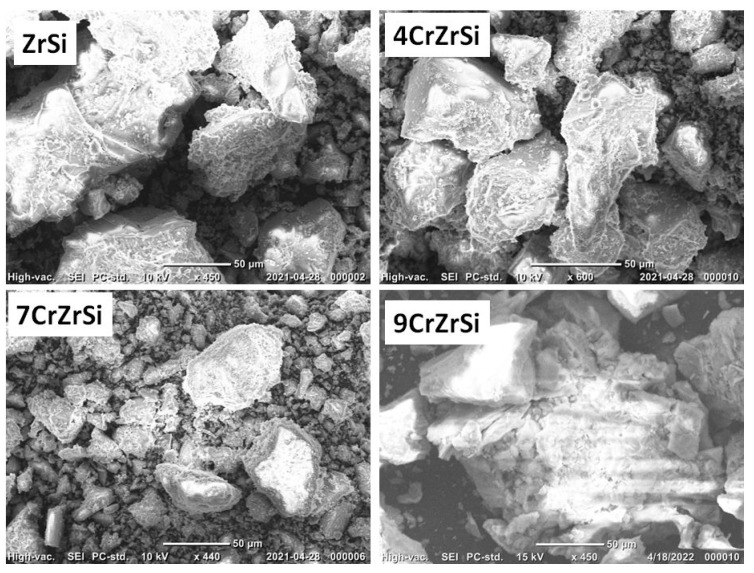
SEM images of fresh ZrSi and CrZrSi catalysts.

The SEM study (Figure 1) demonstrated the similar morphology of all the samples: particles of different sizes and irregular shapes were observed. The SEM-EDX analysis was used to determine the elemental composition of the surface layer of the catalysts. It revealed the presence of small amounts of Br that remained after decomposition of CTAB. The EDX elemental mappings demonstrated the uniform distribution of Zr, Si, and Cr (Figure S1, Supplementary Materials). The EDX surface compositions of the catalysts are presented in Table 1. Higher Cr_2_O_3_ loading in 7CrZrSi than in 4CrZrSi led to higher chromium content on the surface of the former catalyst, while the increase in the Cr_2_O_3_ content up to 9 wt.% did not further enrich the surface with chromium. Therefore, the surface compositions of 7CrZrSi and 9CrZrSi samples were approximately the same.

**Figure 2:**
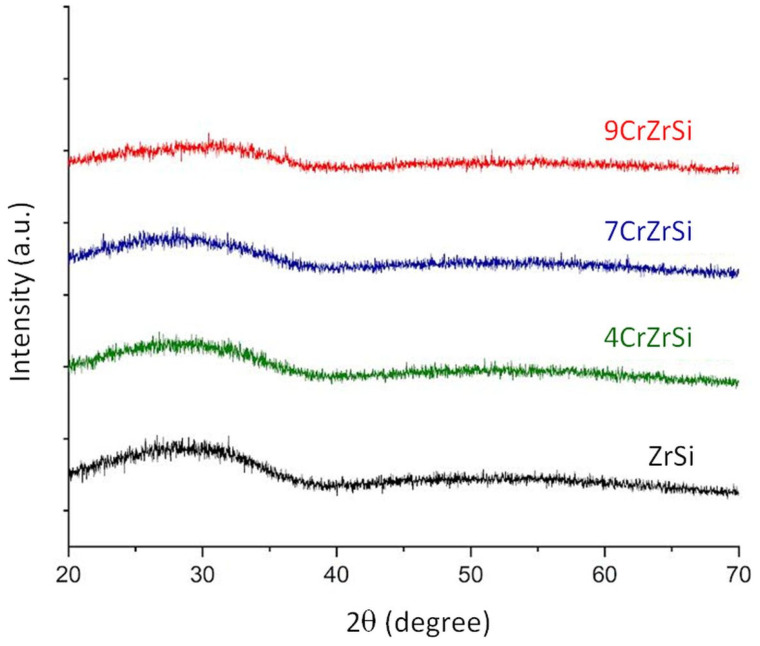
XRD patterns of fresh ZrSi and CrZrSi catalysts.

The XRD analysis revealed the amorphous nature of all the catalysts (Figure 2). Their diffraction patterns comprised only a halo at about 20–35°, typical for amorphous SiO_2_. No reflections of crystalline phases of ZrO_2_, SiO_2_, and chromium oxides were observed in the diffraction patterns. Therefore, only amorphous phases or very small crystallites can exist in the samples. The influence of silica on zirconia crystallization in the mixed ZrO_2_-SiO_2_ samples was reported earlier. Thus, modification of the tetragonal zirconia phase with silica decreased its crystallinity [26,27,28]. SiO_2_ prevented crystallization of ZrO_2_ in the SiO_2_–ZrO_2_ xerogel because the presence of Si–O–Zr bonds retarded the crystal growth upon calcination [29]. So, during the synthesis of our ZrSi and CrZrSi catalysts the structure of the Si-containing framework noticeably increased the rate of ZrO_2_·xH_2_O nucleation and slowed down further growth of nuclei and crystalline phase formation. Therefore, we may expect highly dispersed ZrO_2_ crystallites in the catalysts.

The absence of reflections of CrO_x_ phases testifies to the high dispersion of CrO_x_ in all the samples and indirectly points to the incorporation of chromium ions into zirconia with the formation of a CrZrO_x_ binary solution. The formation of chromium silicates on the surface is also possible [30].

**Figure 3:**
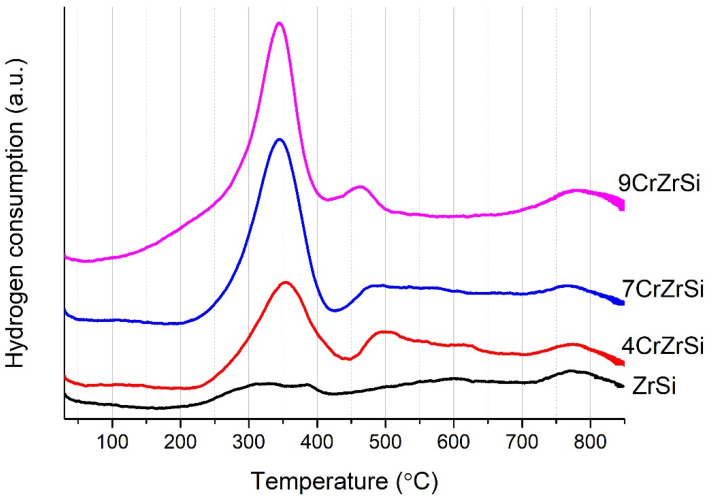
TPR-H_2_ profiles of fresh ZrSi and CrZrSi catalysts.

The bulk chemistry of the metal oxides in the catalysts was analyzed by TPR-H_2_ (Figure 3). This method is sensitive to the electronic state, particle size, component interaction, etc., and it can provide reliable information on the catalyst reducibility. ZrSi showed a very small hydrogen consumption that proceeded in two temperature ranges: from 200 to 400 °C and above 700 °C. The TPR profiles of all the Cr-containing samples also demonstrated a similar high-temperature peak that was obviously related to the reduction of Zr and/or Si species. Although in most cases bulk ZrO_2_ and SiO_2_ are not reduced in the studied temperature range, a small hydrogen uptake above 600 °C can be observed in the TPR-H_2_ profiles of zirconia [31,32].

In the TPR-H_2_ profiles of chromium-containing samples, two additional regions of hydrogen uptake were observed in the temperature ranges of 200–420 °C and 430–500 °C. As hydrogen consumption of ZrSi was very low up to 700 °C, we believe that all intense low-temperatures peaks in the TPR profiles of CrZrSi catalysts are related to the reduction of chromium species.

Large crystalline Cr_2_O_3_ particles cannot be reduced with hydrogen below 800 °C [33]. Therefore, the observed hydrogen consumption peaks can be attributed to the reduction of Cr^6+^ to Cr^3+^ [34]. In particular, the peak at 360 °C can be ascribed to the reduction of CrO_3_ species [34,35]. Hydrogen consumption at about 300 °C corresponds to the reduction of CrO_3_ particles that do not form spinels with the support [36]. The hydrogen uptake in this region can also be attributed to the reduction of non-stoichiometric CrO_x_ species (x > 1.5) [33]. The low-temperature shoulder at 200–300 °C clearly observed in the TPR-H_2_ profile of 9CrZrSi indicates the presence of easily reducible Cr^6+^ species. The high-temperature peak at 450–480 °C is associated with the reduction of highly dispersed Cr_2_O_3_ species [35]; it shifted to lower temperatures in the TPR-H_2_ profile of 9CrZrSi. A broad peak up to 650 °C observed for 4CrZrSi and 7CrZrSi can be attributed to the reduction of non-stoichiometric CrZrO_x_ species formed under Cr_2_O_3_ bonding with ZrO_2_.

The low-temperature hydrogen consumption was quantitatively estimated (Table 1). In contrast, such estimation is difficult for high-temperature peaks due to their significant broadening. The hydrogen consumption corresponded to the peak at 360 °C and increased with increasing Cr content from about 4 to 7 wt.%. Meanwhile, further increase in the chromium content up to 9% did not lead to a significant increase in the hydrogen uptake. This fact was expected and reflects a difference in the content of Cr^6+^ species in the 7CrZrSi and 9CrZrSi samples. It can be assumed that the percentage of Cr^6+^ species decreases when increasing the chromium loading.

Since the PDH reaction proceeds at 500–600 °C, and hydrogen is one of its products, we can expect the reduction of the catalysts under the reaction conditions. According to the TPR-H_2_ data, Cr^6+^ species will be reduced to Cr^3+^ ones under the catalytic test conditions. A fraction of highly dispersed Cr_2_O_3_ species can also be reduced.

**Figure 4:**
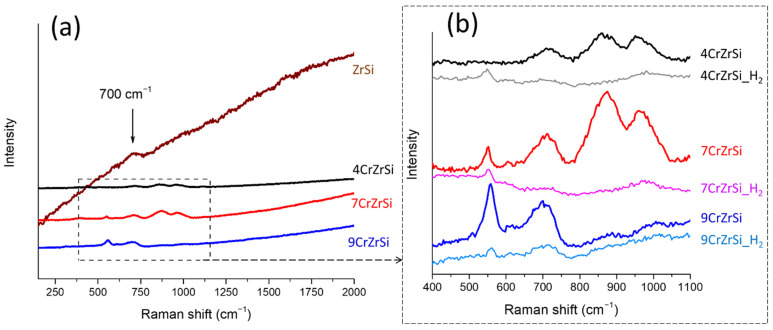
Raman spectra of fresh ZrSi and CrZrSi catalysts (**a**) and the 400–1100 cm^−1^ region of Raman spectra of fresh and H_2_-reduced CrZrSi catalysts (**b**).

The structures of the CrZrSi catalysts were analyzed by Raman spectroscopy. The Raman spectra of ZrSi and the Cr-containing samples are presented in Figure 4. The rise of the background level caused by silica luminescence [37] is especially pronounced for the ZrSi sample. According to the literature data, this effect becomes stronger when increasing the silica dispersion [38]. No lines characteristic for zirconia crystalline phases [39] or SiO_2_ [40] were observed in the spectra. This fact points to the amorphous state of Zr- and Si-containing phases, which agrees with the XRD data. Only a weak signal at about 700 cm^−1^ is visible in the ZrSi spectrum (Figure 4a). Little information can be found in the literature concerning this Raman band. The band of symmetric bending vibrations in Si–O–Si linkages is located at lower wavenumbers of 660 cm^−1^ [41]. A broad band at 700 cm^−1^ in the spectra of crystalline zirconia was attributed to the disorder in the O sublattice induced by the large number of O vacancies [42]. Therefore, a similar band in the Raman spectra of ZrSi points to highly dispersed ZrO_2_ crystallites.

All bands in the Raman spectra of Cr-containing samples (Figure 4b) are broad and poorly resolved, which points to the high structural disorder and/or to the very small size of CrO_x_ crystallites in these materials [43]. This observation agrees with the amorphous nature of the samples detected by XRD.

The band at 700 cm^−1^ was observed not only in the ZrSi Raman spectrum but also in the spectra of Cr-containing samples. The intensity of this band grew when increasing the chromium loading, which can be associated with the increase in the concentration of Zr_cus_^4+^ ions. This growth may also be related to the formation of Cr^3+^ ions octahedrally coordinated with oxygen ([CrO_6_]^9−^). The A_1g_ mode of these species produces a Raman band at about 700 cm^−1^ [44]. The formation of spinel-like structures is highly probable in the “one-pot” synthesis.

The Raman spectrum of the 4CrZrS sample (Figure 4b) also shows two weak and broad lines with maxima at 858 and 960 cm^−1^. These lines are the fingerprint peaks of Cr^6+^ oxide [45,46]. The band at 960–990 cm^−1^ can be attributed to the symmetric stretching vibrations associated with the terminal Cr=O bonds on the surface of chromium oxides [37]. This band points to the presence of isolated surface monochromate species [37]. The absence of Raman bands in the range of 200–300 cm^−1^ also demonstrates that surface CrO_x_ species are mainly isolated [47]. The band at about 860 cm^−1^ can also be attributed to bridging Cr−O−Cr vibrations of a polymeric chromium species [48,49].

The Raman spectrum of 7CrZrSi shows an additional band at 557 cm^−1^. This band can be attributed to the Cr−O−Cr vibrations of Cr^3+^ species in Cr_2_O_3_ [50,51]. The intensities of the bands at 870 and 960 cm^−1^ for 7CrZrSi are higher than those for 4CrZrSi due to the higher chromium content in the former sample. However, a further increase in the chromium content (9CrZrSi) significantly decreased the intensities of bands at 800–1000 cm^−1^. The Raman spectrum of 9CrZrSi clearly shows two pronounced signals at 557 and 700 cm^−1^. The Raman spectra of the Cr-containing catalysts revealed the predominance of Cr_2_O_3_ and Cr^3+^ forms in 9CrZrSi catalyst in contrast to other Cr-containing samples: 4CrZrSi comprised mainly Cr^6+^, while 7CrZrSi comprised both Cr^6+^ and Cr^3+^ species.

To check the possibility of chromium oxide reduction under the PDH reaction conditions, the catalysts after hydrogen treatment at 550 °C for 30 min were also examined by Raman spectroscopy (Figure 4b). The intensities of peaks in the Raman spectra decreased after treatment. The Raman scattering is typically sensitive to the degree of crystallinity of the sample: spectra of crystalline materials show narrow intense peaks, while those of amorphous materials are broader and less intense [52]. Therefore, based on the Raman spectra we can conclude that the reductive treatment did not lead to sintering or crystallization of the catalysts. It mainly resulted in the reduction of Cr^6+^ species as indicated by the nearly complete disappearance of Raman bands at 800–1000 cm^−1^. The spectra of all the reduced catalysts showed a band at 550 cm^−1^ attributed to Cr_2_O_3_. Thus, Cr^6+^ species in the fresh catalysts are easily reduced to Cr^3+^. The band at 700 cm^−1^ was observed in the spectra of all the reduced catalysts, but it has a significant intensity only for 9CrZrSi_H_2_. The presence of this band indicates that the 9CrZrSi catalysts retained the Zr_cus_^4+^ ions or Cr^3+^ species octahedrally coordinated with oxygen even after the reductive treatment. Therefore, the high dispersion of phases in the samples was retained even after the reductive treatment at 550 °C because the “one-pot” synthesis provided the uniform distribution of zirconia and chromium oxides in the fresh catalysts. This fact can be considered as an advantage of the synthesized catalysts.

The surface of the catalysts was studied by XPS. The survey XPS spectra revealed the presence of silicon, zirconium, carbon, oxygen, and chromium (Figure S2, Supplementary Materials). The positions of the main XPS lines are summarized in Table S1 (Supplementary Materials). The binding energies of the Si2p and Zr3d_5/2_ lines for the catalysts are typical for Si^4+^ and Zr^4+^ species but they are intermediate between those of pure SiO_2_ and ZrO_2_ oxides and zirconium silicate (ZrSiO_4_) studied earlier in [53]. This fact points to the interaction between silica and zirconia with the formation of Zr−O−Si bonds.

The Zr/Si molar ratios in the catalysts calculated from the XPS data (Table 1) slightly differ from the values calculated from the SEM-EDX data. Due to the surface sensitivity of XPS, it can be concluded that the surface of 9CrZrSi was slightly enriched with Zr compared to the bulk.

**Figure 5:**
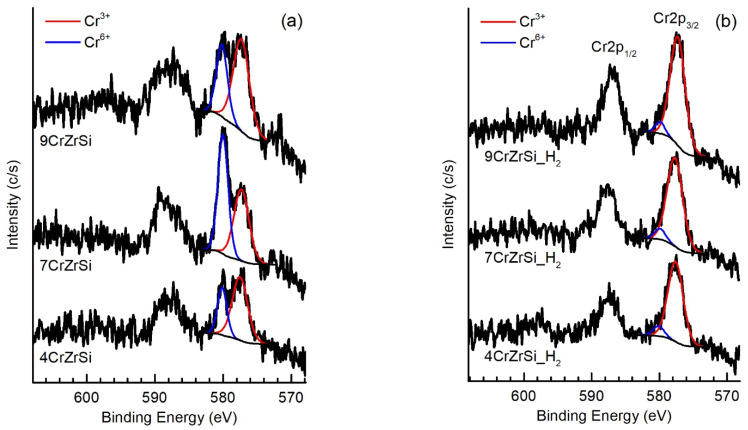
Cr2p XPS spectra of fresh (**a**) and hydrogen-treated (**b**) CrZrSi catalysts. Spectra were recorded in less than 100 s at the beginning of XPS experiment (immediately after X-ray gun and neutralizer were switched on).

The Cr2p XPS spectra of the fresh catalysts showed a pronounced degradation under X-ray radiation (see, for example, the spectra of 7CrZrSi in Figure S3a in the Supplementary Materials) because of the partial reduction of chromium species, which is typical for hexavalent chromium compounds [54]. Due to this fact, the Cr2p spectra recorded within a short acquisition time at the beginning of the XPS experiment were analyzed (Figure 5a). Fitting of these spectra revealed two components with Cr2p_3/2_ binding energies of about 577.5 and 580.0 eV that are typical for Cr^3+^ and Cr^6+^ species, respectively (Table S1, Supplementary Materials). At the same time the Cr2p_3/2_ binding energy of 577.5 eV is higher than that of Cr_2_O_3_ (576.7 eV [55]) and agrees with that of Cr(OH)_3_ (577.5 eV [56]). Higher binding energy of Cr^3+^ species may also result from their coordination with silica and zirconia. The calculated percentages of Cr^3+^ species in the fresh catalysts (Table 2) may be overestimated because of the partial reduction of Cr^6+^ species under X-ray radiation. Nevertheless, because the spectra were acquired in the same conditions, we can compare the percentage of Cr^3+^ species in the catalysts. 7CrZrSi showed the lowest percentage of Cr^3+^ species, while they were about the same in 4CrZrSi and 9CrZrSi. Though the XPS spectra of the catalysts show the presence of Cr^3+^ species, their content can be overestimated because of the X-ray-induced reduction of Cr^6+^ species, and these Cr^3+^ species cannot be assigned to Cr_2_O_3_ oxide because of the higher binding energy of the Cr2p_3/2_ component in the catalyst spectra. This result mostly agrees with the absence of the characteristic bands of bulk crystalline Cr_2_O_3_ oxide in the Raman spectra of the catalysts.

While the bulk chromium loading in the catalysts increased, the Cr/Zr molar ratio on the surface of 7CrZrSi and 9CrZrSi remained about the same but higher than that for 4CrZrSi. This may be caused by chromium incorporation into the silica–zirconia matrix and may affect the formation of Zr_cus_^4+^ on the catalyst surface.

The Cr2p XPS spectra of the reduced Cr-containing catalysts did not show any signs of X-ray-induced degradation (see, for example, the spectra of 7CrZrSi_H_2_ in Figure S3b in the Supplementary Materials). These spectra mainly show the contribution from Cr^3+^ species (Figure 5b). Though fitting of these spectra demonstrated the presence of a small fraction of Cr^6+^ species (6–8%, Table 2), these values are below the detection limit of Cr^6+^ species (10% [56]). Therefore, the XPS data confirm the transformation of Cr^6+^ species into Cr^3+^ ones under reductive treatment at 550 °C, as was proposed from the TPR-H_2_ and Raman spectroscopy data. No significant changes in the surface Cr/Zr molar ratios were observed in the catalysts after H_2_ treatment. This result contradicts the assumption that the decrease in the intensity of Raman spectra after H_2_ treatment is caused by the depletion of the surface with chromium. So, the Cr^6+^ reduction to Cr^3+^ under reductive treatment at 550 °C did not lead to sintering and formation of large crystals.

Thus, the TPR-H_2_, XPS, and Raman spectroscopy study demonstrated that after a short-term reductive treatment all highly oxidized forms of Cr were reduced mainly to Cr^3+^ ones. 9CrZrSi most likely retained a fraction of Cr^3+^ species octahedrally coordinated with oxygen. Thus, under reaction conditions Cr^6+^ species are expected to be reduced with hydrogen produced in the PDH reaction.

### 2.2. Catalytic Tests

The catalysts were tested in non-oxidative PDH at 500–600 °C in a fixed bed flow reactor using a mixture of 40 vol.% of propane and 60 vol.% of nitrogen as a feedstock. ZrSi showed a negligible activity in the PDH reaction at 500 °C and slightly higher activity at 550 and 600 °C (0, 2.0, and 1.2% of propane conversions, respectively). Therefore, further analysis involves only the more active CrZrSi samples.

**Figure 6:**
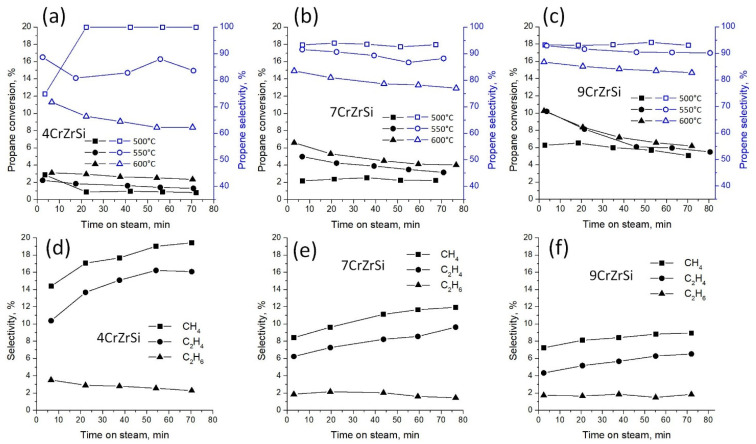
Propane conversion and propylene selectivity (**a**–**c**), and selectivity to by-products at 600 °C (**d**–**f**) over CrZrSi catalysts.

The catalyst efficiencies were compared in terms of the propane conversion, propylene selectivity, and the initial rate of propylene formation. In all experiments, the propane conversion X(C_3_H_8_) (Figure 6a–c) was the highest at the beginning of the test and then slightly decreased, which is typical for the PDH reaction [20]. The decrease in conversion may be caused by the evolution of active sites in the reaction medium or by coke formation that is very likely in the reaction conditions [20]. The DSC-TG analysis of the catalysts after 200 min of the test revealed 5–6 mass. % of coke (Table 3). The coke formation resulted from side reactions. In addition to the main product (propylene), by-products (methane, ethane, and ethylene) were detected in the effluent. The selectivities to by-products are presented in Figure 6d–f. The selectivity to ethane remained unchanged at 500 and 550 °C while those to methane and ethylene simultaneously slowly increased. Therefore, CH_4_ and C_2_H_4_ are formed under hydrogenolysis of C−C bonds in propane. It is important to note that although the propane conversion decreased during time-on-stream, the propylene selectivity remained virtually constant. Only for 9CrZrSi at 550 °C did the S(C_3_H_6_) value decrease but it became constant after 50 min time-on-stream, while at 600 °C a constant although not very strong decrease in S(C_3_H_6_) was observed during the whole catalytic test. The formation and further oligomerization of cracking products decreased propane conversion. Coking is regarded as a cause of catalyst deactivation [4]. Considering the high reaction temperature, sintering is also possible. It is likely that the composition of the active sites did not change much under the action of the reaction mixture since the propylene selectivity remained approximately constant. A strong change in the nature of the active sites would affect the selectivity to a larger extent. The catalyst regeneration experiments confirmed coking to be the main reason for catalyst deactivation. In these experiments, after the catalyst was tested at 600 °C the inlet-gas stream to the reactor was switched from the reaction mixture to air for 10 min. This treatment recovered catalyst activity: after regeneration, the propane conversion reached 93–100% of the initial value (Table 3). The fact that high-temperature reductive treatment did not intensify the bands in the Raman spectra of the catalysts confirmed their stability against sintering.

The modification of ZrSi with chromium and the increase in its loading from 4 to 9 wt.% noticeably enhanced the catalyst performance. The increase in the reaction temperature from 500 to 600 °C increased the propane conversion (Figure 6a–c), but the degree of the increase depended on the chromium content in the catalyst. The effect of the reaction temperature on the propane conversion was more pronounced for 4CrZrSi and 7CrZrSi. The propane conversion over 9CrZrSi was about 6% at 500 °C and about 10% both at 550 and 600 °C. The selectivity to propylene S(C_3_H_6_) strongly (by 40%) decreased for 4CrZrSi with increasing temperature, but less pronounced decreases of 13 and 9% were observed for 7CrZrSi and 9CrZrSi (Figure 6a–c).

The high content of propane in the feed ensured a sufficiently low conversion, which made it possible to analyze the reaction kinetics. The initial rates of propylene formation (Table 3) were calculated with Equation (3) and used to compare the efficiencies of the catalysts with the literature data. As the equilibrium conversion depends on the propane partial pressure in the feed flow, a reliable comparison is possible only with the catalysts tested in similar reaction conditions. The initial rates of propane conversion calculated in our work coincide in order of magnitude with those reported in [20] for the CrZrO_x_ and CrOx/LaZrO_x_ catalysts. The highest initial propane conversion rates of 0.5 and 0.47 mmol (C_3_H_6_)/(g min) observed for 9CrZrSi at 550 and 600 °C, respectively, are only slightly lower than those reported in [20]. The selectivities to propylene over 7CrZrSi and 9CrZrSi are close to those of the commercial K-CrO_x_/Al_2_O_3_ (19.7 wt.% of Cr_2_O_3_) catalyst. Comparing the 7CrZrSi and 9CrZrSi catalysts, we conclude that their selectivities to propylene are approximately the same, while 9CrZrSi demonstrates higher propane conversion and the lowest side-product selectivities, which makes this catalyst more attractive at lower temperatures.

## 3. Discussion

In the present study, two paths were used to improve the efficiency of chromium catalysts in PDH: firstly, the addition of silica provided large specific surface area of the catalyst; secondly, the use of the “one-pot” method for the synthesis of three-component CrO_x_-ZrO_2_-SiO_2_ catalysts improved the distribution of components. Both these modifications contributed to the increase in the dispersion of zirconia and chromium oxides in the catalysts and prevented the formation of low-activity large crystallites.

**Scheme 1:**
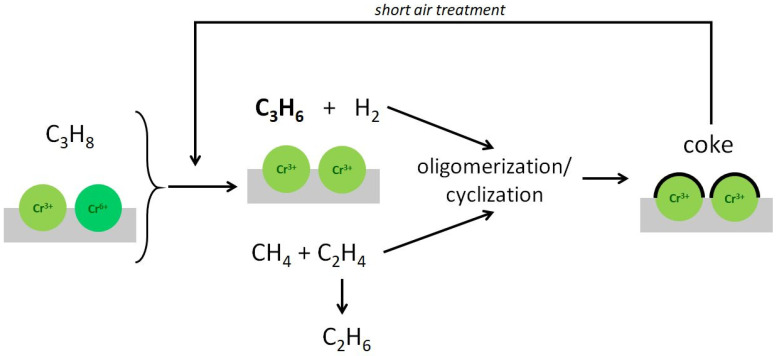
Non-oxidative propane dehydrogenation, side reactions, and changes in chromium oxidation state.

All the Cr-containing catalysts turned out to be active in the PDH reaction. Based on the selectivity data for these catalysts, several conclusions can be drawn. The selectivities to CH_4_ and C_2_H_4_ increased with increasing time-on-stream, which led to catalyst coking. Coking was the main reason for catalyst deactivation because the air treatment recovers catalysts efficiency. By-products were observed in the reaction mixture at low conversions (less than 10%). At such conversion levels, they can be assumed as primary products. The analysis of the selectivity–conversion dependencies (Figure S4, Supplementary Materials) confirms this fact. The CH_4_ and C_2_H_4_ selectivities increase when decreasing the propane conversion. Therefore, the propane dehydrogenation into propylene as a target product and its cracking into CH_4_ and C_2_H_4_ (Scheme 1) proceeded simultaneously. Ethane was formed via hydrogenation of C_2_H_4_. Further oligomerization produced alkenes, and then their cyclization formed coke (Scheme 1).

Another important observation is that only one catalyst (4CrZrSi) exhibited at 500 °C an increase in the propylene selectivity during the catalytic test. This is directly related to the surface composition of the fresh catalysts: 4CrZrSi was the only sample containing no Cr_2_O_3_ according to the Raman spectroscopy data. The observed increase in the propylene selectivity was associated with the reduction of Cr^6+^ to Cr^3+^ under the reaction conditions (Scheme 1). The other two catalysts (7CrZrSi and 9CrZrSi) initially contained Cr_2_O_3_ species, so the selectivity to propylene over these catalysts was high even at the very beginning of the test. Formation of coke decreased propane conversion. The catalyst can be easily regenerated by its short-term exposure to air in the same reactor (Scheme 1).

The initial rates of propylene formation at different temperatures rose when increasing the chromium loading (Table 3). The lowest r_0_(C_3_H_6_) was observed for 4CrZrSi, most likely due to the low content of active Cr^3+^ species on its surface. For 9CrZrSi, the r_0_(C_3_H_6_) values at all temperatures were higher than those for other samples, but they depended little on temperature. r_0_(C_3_H_6_) for 7CrZrSi approached that for 9CrZrSi as the temperature increased up to 600 °C.

The activity of Cr-containing catalysts in PDH is often associated with the presence of highly dispersed α-Cr_2_O_3_, while its large crystallites are inactive [57]. Oxide supports can improve the dispersion of Cr_2_O_3_, making crystallites smaller. Additionally, the redox species provided by the ZrO_2_ or Al_2_O_3_ support are expected to play an important role [20,23,58]. Coordinatively unsaturated Zr^4+^ sites in the CrO_x_/ZrO_2_ catalysts can serve as additional active centers providing synergistic action of Cr and Zr in non-oxidative PDH [20,21]. For example, a synergistic effect of CrO_x_ and coordinatively unsaturated Zr^4+^ centers was observed in the CrO_x_/LaZrO_x_ catalyst in PDH [20]. It was proposed that ZrO_2_-based catalysts (LaZrO_x_ or YZrO_x_) have their own catalytic activity in PDH due to the combination of coordinatively unsaturated zirconium cations (Zr_cus_^4+^) and neighboring lattice oxygen [21]. Zr-C_3_H_7_ and O–H species are formed after the breakage of C–H bonds in C_3_H_8_. At the next stage, hydrogen is desorbed from the surface to form propylene.

Coordinatively unsaturated zirconium cations were observed on the surface of the Ru-modified LaZrO_x_ or YZrO_x_ catalysts due to hydrogen activation on Ru particles [21]. The drawback of the catalysts modified with noble metals is the in situ over-reduction of their surface under the PDH reaction conditions. The intrinsic activity of the support containing Zr_cus_^4+^ sites can increase when increasing their degree of unsaturation [21].

The activity of the CrZrSi catalysts synthesized in the present work is very likely related to the synergistic action of Cr^3+^ and Zr^4+^ sites. According to the XRD data, all the CrZrSi samples were X-ray amorphous, that is, they contained either amorphous phases or very small crystallites. As was discussed above, SiO_2_ is not only amorphous itself, but it also stabilizes amorphous zirconia and possibly chromium oxides. Interaction between the precursors of Si and Zr oxides during “one-pot” co-precipitation inhibits zirconia crystallization [29]. In addition, the formation of Si–O–Zr bonds suggested based on the XPS data probably contributed to the stabilization of Cr-containing active sites. The surface of SiO_2_ modified with zirconia contains ≡Zr−O^−^ sites [13,59]. Acting as anchoring centers, they can suppress crystallization of Cr_2_O_3_ in the CrZrSi samples and prevent formation of large α-Cr_2_O_3_ crystallites that have low activity in PDH [19].

The XPS and Raman data revealed the difference in the surface composition of our Cr-containing catalysts. 4CrZrSi with low chromium loading contained amorphous Cr^3+^ species and Cr^6+^ sites, possibly in CrZrO_x_ formed under interaction of chromium with zirconia. Under H_2_ treatment, the latter sites are reduced to highly dispersed Cr_2_O_3_ species active in PDH. The fresh 7CrZrSi and 9CrZrSi catalysts with higher chromium loadings contain not only Cr^6+^ but also highly dispersed Cr^3+^ species that are relatively resistant to reduction. These Cr^3+^ species significantly change neither their oxidation state nor dispersion in the reductive conditions. Thus, the 7CrZrSi and 9CrZrSi catalysts are more efficient in PDH.

After H_2_ reduction, the Raman band at 700 cm^−1^ that can be attributed to Zr_cus_^4+^ or Cr^3+^ ions octahedrally coordinated with oxygen remained only in the spectrum of 9CrZrSi. Although Cr^6+^ on the surface of the catalysts was totally reduced during short H_2_ treatment, it seems that the optimal combination of Zr_cus_^4+^ and Cr^3+^ species was achieved only in 9CrZrSi. This result indicates that the evolution of the 9CrZrSi catalyst under reaction conditions should proceed according to the most favorable for the PDH route.

Thus, zirconia and silica play an important role in the active site formation. The “one-pot” synthesis makes it possible to produce CrZrSi catalysts with large specific surface area and chromium active centers highly dispersed over the catalyst surface. The co-precipitation with silicon oxide suppresses ZrO_2_ crystallization. The ≡Zr−O^−^ centers formed during co-precipitation apparently participate in the formation of one of the types of active centers, namely coordinatively unsaturated Zr^4+^ ions. Thus, the “one-pot” synthesis provides the formation of both Zr_cus_^4+^ and active Cr^3+^ sites in the CrZrSi catalysts. In the case of low chromium content (4CrZrSi), mainly Cr^6+^ species exist on the surface of the fresh catalyst. These sites are reduced under reaction conditions forming large Cr_2_O_3_ particles with low activity in PDH. In 7CrZrSi and 9CrZrSi, the active Cr^3+^ sites already exist on the surface of the fresh catalysts, providing high catalyst efficiency. The Zr_cus_^4+^ sites in 9CrZrSi are stable and remain on the surface after the reductive treatment. Catalytically active sites comprise the combination of Zr_cus_^4+^ and Cr^3+^ species in a highly dispersed oxide phase. Thus, it is likely that tuning the chromium content in the range from 7 to 9 wt.% of Cr_2_O_3_ is important for increasing the catalyst efficiency. The activities of 7CrZrSi at 600 °C and 9CrZrSi at 500 °C are similar, so 9CrZrSi is more efficient at lower temperatures.

## 4. Materials and Methods

### 4.1. Catalyst Preparation

ZrO_2_-SiO_2_ (further denoted as ZrSi) and CrO_x_-ZrO_2_-SiO_2_ (further denoted as yCrZrSi, where y is the Cr_2_O_3_ loading in wt.%) were synthesized by the template-assisted evaporation-induced self-assembly method (EISA). ZrO(NO_3_)_2_·H_2_O (99.5%, Acros Organics, Geel, Belgium), Cr(NO_3_)_3_·9H_2_O (99.9%, Sigma-Aldrich, St. Louis, MO, USA), cetyltrimethylammonium bromide (CTAB, 99%, BioChemica, Billingham, UK), tetramethylammonium hydroxide (TMAOH, 25% aq.solution, Acros Organics, Geel, Belgium), and tetraethyl orthosilicate (TEOS, reagent grade, Sigma-Aldrich) were used as raw materials for the synthesis. The degree of salt hydration was controlled by DSC-TG. The (Cr + Zr):Si molar ratio in all the samples was 0.8. Chromium content was 0; 4.47; 6.89; and 9.28 wt.% assuming Cr_2_O_3_ stoichiometry for the chromium oxide.

Synthesis of ZrSi. To produce Zr-precursor, the required amount of ZrO(NO_3_)_2_ H_2_O was dissolved in 50 mL of distilled water and added dropwise to a solution of 7.29 g of CTAB in 250 mL of distilled water. The resulting mixture was left for 2 days at room temperature. After that, it was kept in an oven at 95 °C for 48 h. The resulting bright yellow powder was dissolved with stirring in 40 mL of distilled water until a homogeneous suspension was formed. To obtain a silicon precursor, 12 mL of TEOS was added dropwise to 18 mL of aqueous TMAOH solution under stirring. The final clear solution was slowly added dropwise with stirring to the Zr-precursor suspension, and stirred at RT for 2 h to form a white precipitate. It was filtered, dried in air at 90 °C for 48 h, ground to a powder, and calcined in air at 600 °C for 5 h.

CrZrSi samples were prepared by the same method. The required amount of Cr(NO_3_)_3_·9H_2_O dissolved in 55 mL of distilled water was mixed with the ZrO(NO_3_)_2_·H_2_O aqueous solution and added to the CTAB solution.

The CrZrSi-H_2_ samples were obtained by the treatment of the CrZrSi samples with hydrogen at 550 °C in a catalytic reactor. For this purpose, 150 mg of the sample was heated up to 550 °C in a flow of N_2_ (20 mL/min) and then H_2_ (10 mL/min) was passed through reactor for 30 min. Then, the catalyst was cooled in N_2_ flow to RT.

### 4.2. Catalyst Characterization

Chromium content in the samples was measured by atomic absorption spectroscopy (AAS) on a Thermo Fisher Scientific series iCE 3000 spectrophotometer (Thermo Scientific, Waltham, MA, USA) using air–acetylene flame atomization. Solid samples were dissolved in HF (chemically pure, Reachim). The SOLAAR Data Station software was used for the device control and data processing.

XRD diffractograms of the catalysts were recorded on a Rigaku Ultima IV powder diffractometer (Rigaku, Tokyo, Japan) (CuKα radiation, 1.5418 Å) in the 2θ range from 5 to 90° with a step size of 0.02°. The phase composition was analyzed by comparison with the JCPDS PDF1 library data (ICDD database).

X-ray photoelectron spectroscopy (XPS) was used to reveal the composition and oxidation state of elements on the catalyst surface. The spectra were acquired on an Axis Ultra DLD spectrometer (Kratos Analytical, Manchester, UK) with a monochromatic Al*K*_α_ radiation source (hν = 1486.7 eV, 150 W). The pass energies of the analyzer were 160 eV for survey spectra and 40 eV for high-resolution scans. The binding energy scale of the spectrometer was preliminarily calibrated using the position of the peaks for the Au 4*f*_7/2_ (83.96 eV), Ag 3*d*_5/2_ (368.21 eV), and Cu 2*p*_3/2_ (932.62 eV) core levels of pure metallic gold, silver, and copper. The powder samples were fixed on a holder using a double-sided non-conductive adhesive tape. The Kratos charge neutralizer system was used, and the spectra were charge-corrected to give the C1s peak of adventitious carbon a binding energy of 285.0 eV. Due to the observed reduction of chromium species under an X-ray beam, each XPS experiment started and ended with the fast (about 100 s) acquisition of the Cr2p spectrum. The spectra were fitted in the CasaXPS software.

Raman spectra were recorded on a Horiba JobinYvon LabRAM HR 800 UV instrument. An argon ion laser with a wavelength of 514 nm was used for excitation, and the power on the sample did not exceed 7 mW. For each sample, the spectra were accumulated for 200 s.

Scanning electron microscopy (SEM) of the catalysts was performed on a JCM–6000 Neoscope microscope (JEOL Ltd., Tokyo, Japan) equipped with an energy-dispersive X-ray spectroscopy (EDX) accessory.

Nitrogen physisorption isotherms were recorded on an Autosorb–1 instrument (Quantachrome Instruments, Boynton Beach, FL, USA). Before measurement, the samples were outgassed for 3 h at 200 °C. The specific surface areas of the catalysts were calculated by the BET method. Desorption branches of the isotherms were used for the calculation of pore size distributions by the BJH method.

Temperature-programmed reduction with hydrogen (TPR-H_2_) was carried out on a USGA-101 chemisorption analyzer (Unisit, Moscow, Russia). A 5% H_2_/Ar mixture was fed to a quartz reactor at a flow rate of 30 mL/min. The sample weight was approximately 50 mg. Before analysis, a catalyst was preliminarily kept at 300 °C for 30 min in argon flow and then cooled to 30 °C. TPR profiles were recorded when heating the sample from 30 to 900 °C at a rate of 10 °C/min. Hydrogen consumption during analysis was registered with a thermal conductivity detector preliminary calibrated by NiO reduction.

An STA 449C Jupiter thermal analyzer (Netzsch, Selb, Germany) was used for DSC-TG study. The sample was heated from 40 to 900 °C at a rate of 10 °C/min in an alumina crucible in a mixture of air (80 mL/min) and argon (40 mL/min).

### 4.3. Catalytic Tests

The catalysts were tested in oxidative PDH in a ULKat-1 catalytic flow unit (UNISIT, Moscow, Russia) equipped with a fixed bed quartz reactor, a three-zone tubular furnace, a thermocouple, and temperature and flow-mass controllers. The system was controlled using the “CatUnit” software. The pretreatment and test conditions were selected based on the literature data [20]. In all tests, 100 mg of the catalyst (250–500 μm fraction) was used. The catalysts were tested at three temperatures of 500, 550, and 600 °C and at a total pressure of 1 bar. The catalyst was preheated to a reaction temperature in a nitrogen flow (20 mL/min) at a heating rate of 10°/min. After that, the reactor was purged with the reaction mixture of 40 vol.% C_3_H_8_ and 60 vol.% N_2_ (F_0_ = 30 mL/min).

The reaction mixture was fed into the GC column using a six-way valve. GC analysis was performed on a Crystal-5000.2 chromatograph (Chromatec, Yoshkar-Ola, Russia) equipped with an Al_2_O_3_ “S” PLOT capillary column (30 m, id 0.53 mm, “HP”, Santa Clara, CA, USA) and a flame ionization detector. The error in chromatographic area determination is 1%. Propane conversion and product selectivity were calculated using the internal normalization method with response factors:

(1) $$X\left(C_{3}H_{8}\right),\%=\frac{\sum_{j,\mathrm{products}}f\left(j\right)\cdot A\left(j\right)}{\sum_{j,\mathrm{products}}f\left(j\right)\cdot A\left(j\right)+f\left(C_{3}H_{8}\right)\cdot A\left(C_{3}H_{8}\right)}\times 100,$$

(2) $$S\left(i\right),\%=\frac{f\left(i\right)\cdot A\left(i\right)\times 100}{\sum_{j,\mathrm{products}}f\left(j\right)\cdot A\left(j\right)},$$

where A(i) and A(j) are the areas of chromatographic peaks; f(i) and f(j) are calibration factors for the reaction products.

Propylene formation rate was calculated assuming that at conversions below 15% the flow reactor could be considered as a differential type:

(3) $$r\left(C_{3}H_{6}\right)=\frac{F_{0}\cdot X_{C_{3}H_{6}}}{V_{m}\cdot m_{\mathrm{cat}}},$$

where F_0_ is a volumetric feed flow rate of propane (mL/min), $X_{C_{3}H_{6}}$ is a molar fraction of C_3_H_6_, V_m_ is the molar volume (22,400 mL/mol), m_cat_ is the catalyst loading in the reactor (g). The reaction mixture was diluted with nitrogen to eliminate the influence of the pressure drop from the formation of gaseous products on the reaction rate. The absence of mass transfer limitations was also checked.

The catalysts were regenerated in the same reactor at 600 °C immediately after PDH tests by switching the inlet-gas stream from the reaction mixture to air for 10 min. After that, the reactor was purged with nitrogen for 5 min and the inlet-gas stream was switched back to the reaction mixture.

The degree of propane conversion recovery was calculated as:

(4) $$R_{X\left(C_{3}H_{6}\right)}=\frac{X_{C_{3}H_{6}}}{X_{C_{3}H_{6}\left(\mathrm{air}\right)}}\cdot 100\%,$$

where $X_{C_{3}H_{6}}$ and $X_{C_{3}H_{6}\left(\mathrm{air}\right)}$ are, respectively, propane conversions in the first run and after regeneration.

## 5. Conclusions

The one-pot template-assisted evaporation-induced self-assembly synthesis provided the high dispersion of catalytically active sites in the CrZrSi catalysts. Silica played a crucial role of textural promoter in the catalysts. Co-precipitation led to the formation of Si−O−Zr bonds, which resulted in the high dispersion of zirconia with the formation of coordinatively unsaturated Zr^4+^ centers that improved the catalyst efficiency. Silica also prevented over-crystallization of Cr_2_O_3_, improving its catalytic action. Chromium in fresh 4CrZrSi existed as amorphous Cr^3+^ and highly dispersed Cr^6+^ species that transformed to Cr^3+^ ones under the reductive reaction conditions. However, these sites had low activity in PDH due to the unfavorable structure of the phase in which they are located. The 7CrZrSi and 9CrZrSi catalysts with higher chromium loadings initially contained dispersed crystalline Cr^3+^ sites that are more resistant to aggregation under the reaction conditions. That is why these catalysts are more efficient in PDH. The propane conversions and propylene selectivities were similar for 7CrZrSi (at 600 °C) and 9CrZrSi (at 500 °C). So, 9CrZrSi was more efficient at 500 °C, probably due to the close to optimal Zr_cus_^4+^ to Cr^3+^ ratio in this catalyst. The results of this work demonstrate that the “one-pot” synthesis and addition of silica modifier make it possible to synthesize efficient catalysts with a relatively low chromium content. Sintering resistance of highly dispersed Cr-containing particles at high temperatures is an advantage of the catalysts produced by the “one-pot” synthesis.

## Figures and Tables

**Table 1:** Physicochemical parameters of fresh catalysts.

Sample	Cr_2_O_3_, wt.% (AAS)	S_BET_, m^2^/g	SEM-EDX		XPS			H_2_ Uptake ^1^, μmol/g
			Cr, wt.%	Zr:Si	Cr, wt.%	Cr/Zr	Zr:Si	
ZrSi	–	464 ± 46	0	1:2.1	0	–	1:2.5	–
4CrZrSi	4.47 ± 0.03	517 ± 52	1.4	1:2.1	3.2	0.19	1:2.4	227
7CrZrSi	6.89 ± 0.07	518 ± 52	2.9	1:2.4	4.2	0.28	1:2.7	357
9CrZrSi	9.28 ± 0.07	443 ± 44	2.7	1:2.3	3.8	0.28	1:3.5	381

^1^ Measured by hydrogen consumption during TPR-H_2_ in temperature range of 200–420 °C.

**Table 2:** The percentage of Cr^3+^ species relative to the total Cr content and Cr/Zr molar ratio in fresh and hydrogen-treated CrZrSi catalysts determined by XPS.

Sample	Cr^3+^, %		Cr/Zr	
	Fresh	H_2_ Treated	Fresh	H_2_ Treated
4CrZrSi	69	92	0.19	0.18
7CrZrSi	47	92	0.27	0.24
9CrZrSi	65	94	0.28	0.26

**Table 3:** The initial rates of propylene formation (r_0_(C3H6)) for ZrSi and CrZrSi catalysts and degree of propane conversion recovery ($R_{X\left(C_{3}H_{6}\right)}$).

Scheme 1.	Coke Content in Used Catalyst ^1^, %	r_0_(C_3_H_6_) (mmol(C_3_H_6_)g^−1^min^−1^)			$R_{X\left(C_{3}H_{8}\right)}$^2^, %
		500 °C	550 °C	600 °C	
ZrSi	–	—	0.01	0.03	—
4CrZrSi	6.2	0.11	0.11	0.12	—
7CrZrSi	5.9	0.11	0.24	0.29	100
9CrZrSi	5.1	0.31	0.50	0,47	93

^1^ Calculated by weight loss during DSC-TG analysis of used catalyst (200 min in PDH test); ^2^ degree of C_3_H_8_ conversion recovery after 10 min air treatment of used catalyst at 600 °C.

## Data Availability

Not applicable.

## Supplementary Materials

The following supporting information can be downloaded at: https://www.mdpi.com/article/10.3390/molecules27186095/s1, Figure S1: SEM images and SEM-EDX elemental mappings of fresh CrZrSi catalysts; Figure S2. Survey XPS spectra of fresh ZrSi and CrZrSi catalysts; Figure S3. Cr2p XPS spectra of fresh (A) and hydrogen-treated (B) 7CrZrSi catalyst; Figure S4. CH_4_, C_2_H_4_ and C_2_H_6_ selectivities vs propane conversion during PDH at 600 °C on 9CrZrSi; Table S1. Binding energies of XPS peaks for the fresh and hydrogen-treated ZrSi and CrZrSi catalysts and reference compounds.

## Sample Availability

Samples of the catalysts are not available from the authors.
