# Supplementary material for: Non-Oxidative Propane Dehydrogenation on CrOx-ZrO2-SiO2 Catalyst Prepared by One-Pot Template-Assisted Method
Source: Molecules. 2022 Sep 18;27(18):6095. doi: 10.3390/molecules27186095 (PMC9501860; doi:10.3390/molecules27186095)
Supplement: Supplementary file 1 [file molecules-27-06095-s001.zip › molecules-1893762-supplementary.pdf]

Supplementary materials for article

Non-oxidative propane dehydrogenation on CrO_x-ZrO_2-SiO_2 catalyst prepared by one-pot template-assisted method

Elena V. Golubina, Igor Yu. Kaplin, Anastasia V. Gorodnova, Ekaterina S. Lokteva, Oksana Ya. Isaykina, Konstantin I. Maslakov

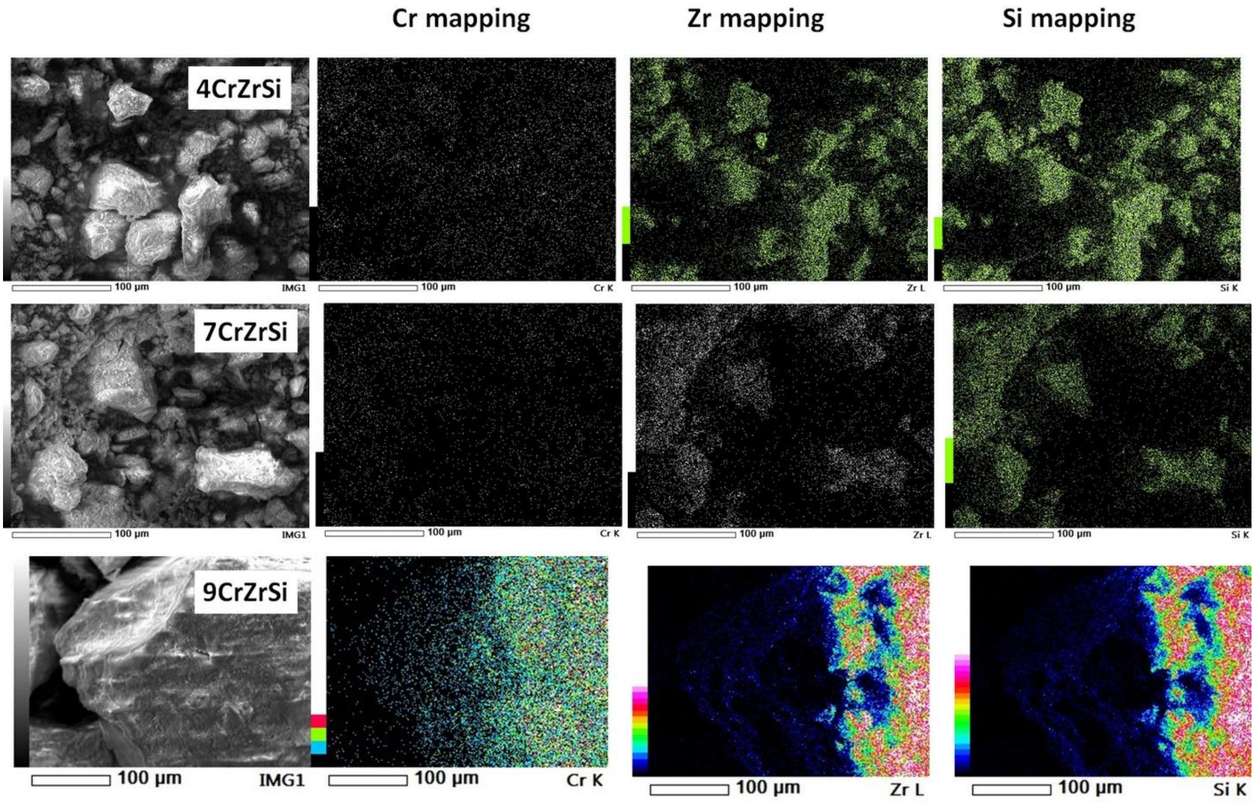

Figure S1. SEM images and SEM-EDX elemental mappings of fresh CrZrSi catalysts.

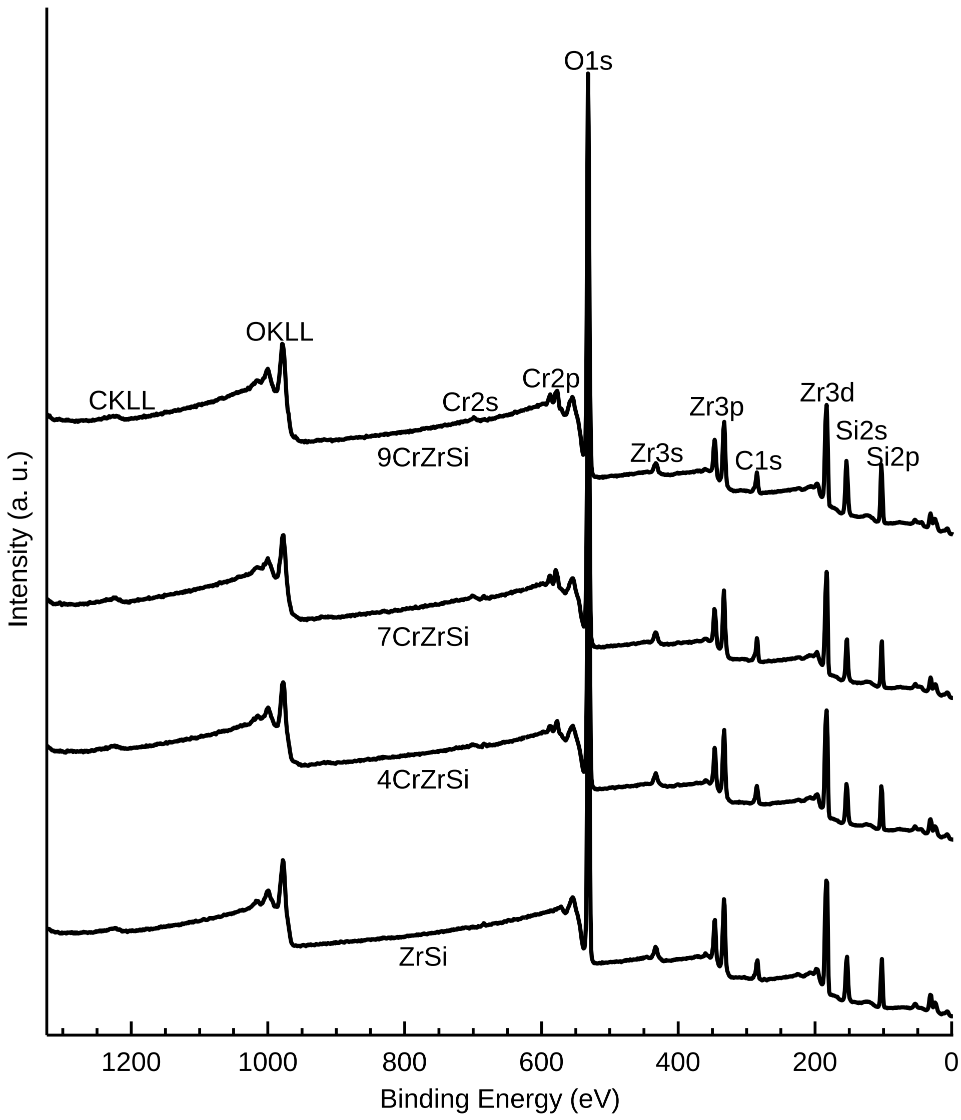

Figure S2. Survey XPS spectra of fresh ZrSi and CrZrSi catalysts

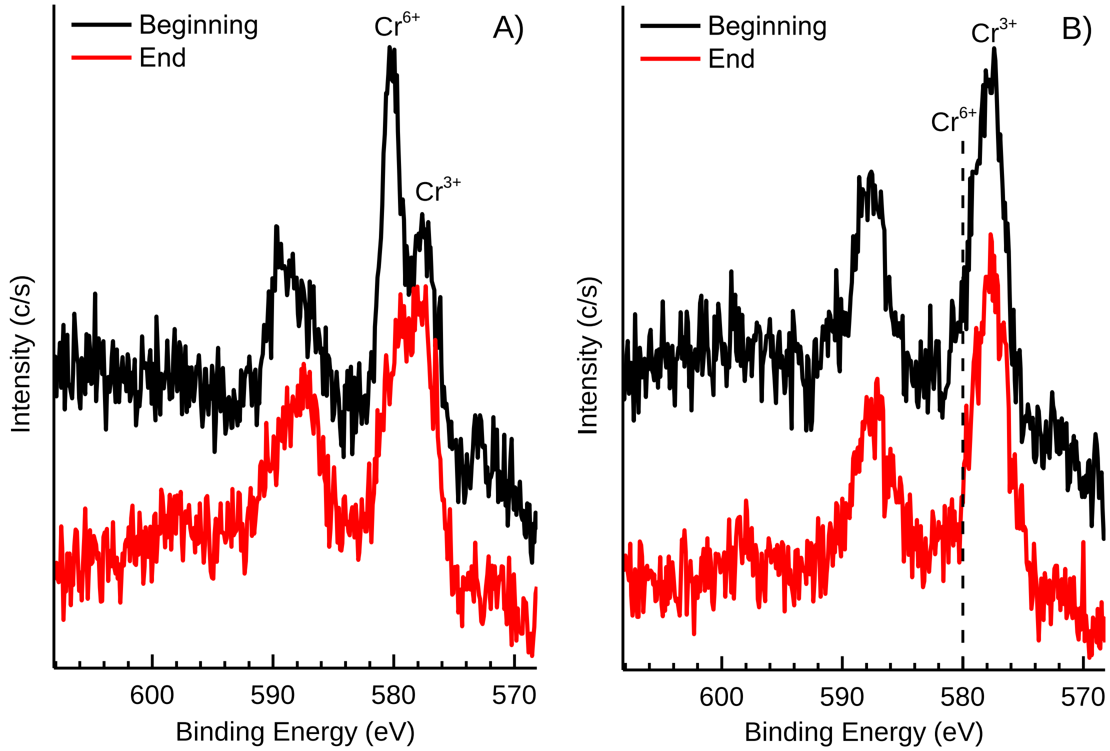

Figure S3. Cr2p XPS spectra of fresh (A) and hydrogen treated (B) 7CrZrSi catalyst. Spectra were recorded in less than 100 s at the beginning (immediately after X-ray gun and neutralizer were switched on) and at the end of XPS experiment.

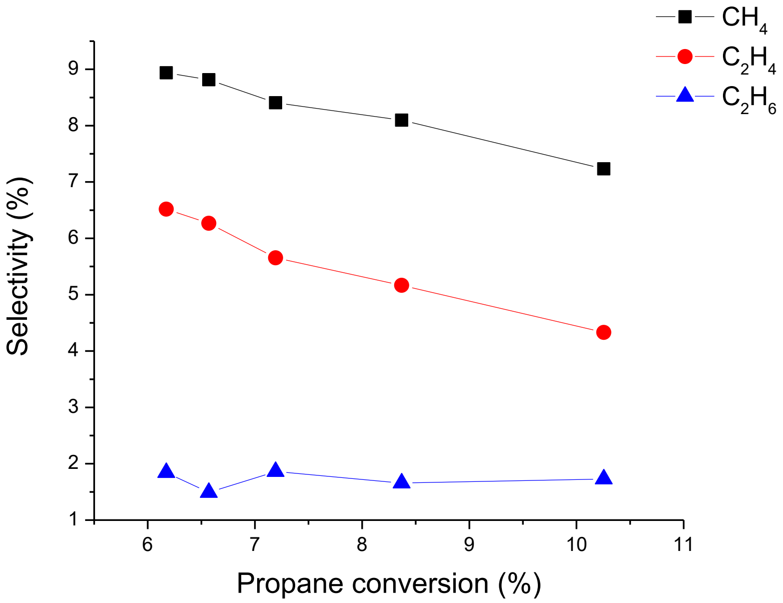

Figure S4. CH_4, C_2H_4 and C_2H_6 selectivities vs propane conversion during PDH at 600°C on 9CrZrSi

Table S1. Binding energies of XPS peaks for the fresh and hydrogen treated ZrSi and CrZrSi catalysts and reference compounds. Both experimental and literature binding energies were corrected to C1s peak at 285.0 eV.

Sample	Si2p	$Zr3d_{5/2}$	$Cr2p_{3/2}$		C1s	Reference
			Cr^{3+}	Cr^{6+}		This work
ZrSi	103.2	183.1			285.0	This work
4CrZrSi	103.1	183.0	577.5	580.1	285.0	This work
7CrZrSi	102.9	182.9	577.3	580.0	285.0	This work
9CrZrSi	103.3	182.9	577.3	580.2	285.0	This work
4CrZrSi_H2	102.9	182.9	577.6	580.1	285.0	This work
7CrZrSi_H2	103.2	183.0	577.7	579.9	285.0	This work
9CrZrSi_H2	103.5	183.1	577.3	579.9	285.0	This work
ZrO_2		182.5			285.0	[1]
SiO_2	103.0				285.0	[1]
$ZrSiO_4$	103.4	183.6			285.0	[1]
Cr_2O_3			576.7		285.0	[2]
$Cr(OH)_3$			577.5		285.0	[3]
CrO_3				579.8	285.0	[3]

References:

1. Dementjev A.P., Ivanova O.P., Vasilyev L.A., Naumkin A.V., Nemirovsky D.M., Shalaev D.Y. Altered layer as sensitive initial chemical state indicator* // Journal of Vacuum Science & Technology A. 1994. V. 12. № 2. P. 423-427.
2. Biesinger M.C., Brown C., Mycroft J.R., Davidson R.D., McIntyre N.S. X-ray photoelectron spectroscopy studies of chromium compounds // Surface and Interface Analysis. 2004. V. 36. № 12. P. 1550-1563.
3. Biesinger M.C., Payne B.P., Grosvenor A.P., Lau L.W.M., Gerson A.R., Smart R.S.C. Resolving surface chemical states in XPS analysis of first row transition metals, oxides and hydroxides: Cr, Mn, Fe, Co and Ni // Applied Surface Science. 2011. V. 257. № 7. P. 2717-2730.
